# ACETABULAR RECONSTRUCTION USING CUP-ON-CUP AND CUP-CAGE TECHNIQUES IN PAPROSKY IIC, IIIA, AND IIIB DEFECTS: A CASE SERIES WITH CLINICAL AND RADIOGRAPHIC EVALUATION

**DOI:** 10.1590/1413-785220263406e310580

**Published:** 2026-11-09

**Authors:** Thiago Augusto da Silva, Rafael Santiago, Giovani Pinoti, Sttéphani Campos de Melo, Francisco Fontes Cintra, Rodrigo Gonçalves Pagnano

**Affiliations:** 1 Universidade Estadual de Campinas, UNICAMP, Campinas, SP, Brazil.

**Keywords:** Acetabulum, Arthroplasty, Replacement, Hip, Hip Joint, Arthroplasty, Replacement, Acetábulo, Artroplastia de Quadril, Articulação do Quadril, Artroplastia de Substituição

## Abstract

**Purpose:**

To describe the clinical and radiographic outcomes of the cup-on-cup and cup-cage techniques in Paprosky IIC, IIIA, and IIIB acetabular defects in hip revision surgery.

**Patients and methods:**

Retrospective case series involving 13 patients who underwent acetabular component revision using cup-on-cup (n=7) or cup-cage (n=6), with defects classified as Paprosky IIC, IIIA, or IIIB and minimum follow-up of 24 months. The factors evaluated were range of motion, flexion strength, claudication, dysmetria, and radiographic restoration of the hip center of rotation.

**Results:**

Mean age was 67.5 ± 12.0 years and mean follow-up 54.2 ± 33.6 months. Acetabular osteolysis was present in 76.9% of cases. Center of rotation restoration was achieved in 66.7% of cup-cage cases and 42.9% of cup-on-cup group. Mean active flexion was 91.7° and 85.7°, respectively. One dislocation (7.7%) was treated with closed reduction. No periprosthetic infection or reoperation was recorded.

**Conclusions:**

Both techniques demonstrated satisfactory outcomes in severe acetabular defects, with no major complications. The choice of techniques should be individualized based on the severity of the defect, the surgeon's experience, and the availability of the implant. *Level of Evidence IV; Case Series.*

## RESUMO

**Objetivo:**

Descrever os resultados clínicos e radiográficos das técnicas cup-on-cup e cup-cage em defeitos acetabulares Paprosky IIC, IIIA e IIIB na cirurgia de revisão da artroplastia de quadril.

**Pacientes e métodos:**

Série de casos retrospectiva envolvendo 13 pacientes submetidos à revisão do componente acetabular utilizando as técnicas cup-on-cup (n=7) ou cup-cage (n=6), com defeitos classificados como Paprosky IIC, IIIA ou IIIB e seguimento mínimo de 24 meses.

**Foram avaliados:**

amplitude de movimento, força de flexão, claudicação, dismetria e restauração radiográfica do centro de rotação do quadril.

**Resultados:**

A média de idade foi de 67,5 ± 12,0 anos e o tempo médio de seguimento foi de 54,2 ± 33,6 meses. Osteólise acetabular estava presente em 76,9% dos casos. A restauração do centro de rotação foi alcançada em 66,7% dos casos do grupo cup-cage e em 42,9% do grupo cup-on-cup. A flexão ativa média foi de 91,7° e 85,7°, respectivamente. Um caso de deslocamento (7,7%) foi tratado com redução fechada. Não foram registrados casos de infecção periprotética ou reoperação.

**Conclusões:**

Ambas as técnicas demonstraram resultados satisfatórios em defeitos acetabulares graves, sem complicações maiores. A escolha da técnica deve ser individualizada com base na gravidade do defeito, na experiência do cirurgião e na disponibilidade do implante. *Nível de Evidência IV; Série de Casos.*

## INTRODUCTION

Total hip arthroplasty is recognized as one of the surgical procedures with the greatest impact on the quality of life of patients with degenerative joint disease. Projections predict a 137% increase in the volume of revision surgeries in the United States between 2005 and 2030.^1^ Regarding revisions, acetabular reconstruction represents one of the greatest technical challenges in orthopedics, particularly when associated with extensive bone loss resulting from aseptic osteolysis, implant loosening, or septic failure.^2-4^

Several classification systems have been proposed to stratify acetabular defects. One of the main ones is the Paprosky system, proposed in 1994, which remains the most widely used for evaluating acetabular defects in revision surgeries. It classifies bone loss based on location, extent of the defect, and integrity of the acetabular columns. ^5-7^ Defects of types IIC, IIIA, and IIIB represent a high degree of reconstruction complexity, characterized by a superiorly migrated center of rotation, extensive ischiatic osteolysis, compromise of the Köhler line, and bone-to-implant contact of less than 50% of the available acetabular surface.^4,7,8^ The lack of consensus regarding the best surgical technique to be used in the revision of acetabular defects reflects the challenge, the surgical difficulty, and the diversity of available implants.^9^

Several techniques have been used to manage severe acetabular defects, including *jumbo cups*, ultra-porous cups, wedges/porous metal blocks, structural allografts, customized triflanged implants (CTAC), cup-on-cup and cup-cage techniques.^4,7-11^ In defects classified as type III without pelvic discontinuity, the combination of a porous hemispherical cup with trabecular metal wedges currently constitutes the gold standard, with survival rates exceeding 90% at ten years. ^7,12^ In defects with pelvic discontinuity or in those where stable primary fixations cannot be achieved with a dome and wedges alone, the cup-cage technique (Figure 1, 2a and 2b) appears to be the alternative offering greater immediate stability.^7,9,13,14^ Systematic reviews show an overall revision rate of 8% and a complication rate of 20% for this technique. Dislocation is the most frequent complication.^14^ Series with a mean follow-up exceeding ten years report a 90% survival rate without failures due to aseptic loosening. These data corroborate the concept that the technique's success results from the combination of immediate mechanical stability and progressive biological integration, regardless of the material used.^15^

**Figure 1:**
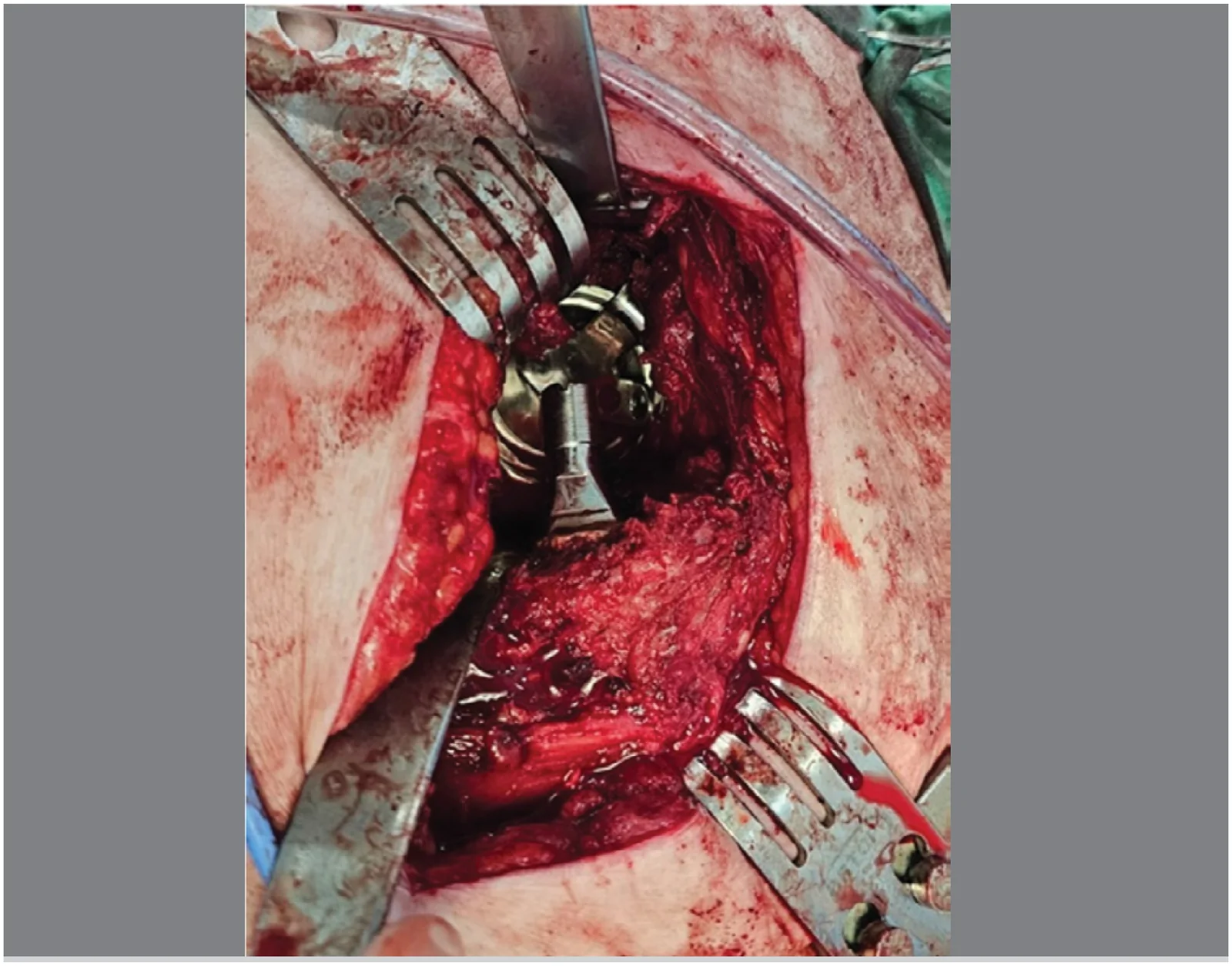
Intraoperative image of a cup-cage surgery technique.

Original image from the authors’ institution.

**Figure 2:**
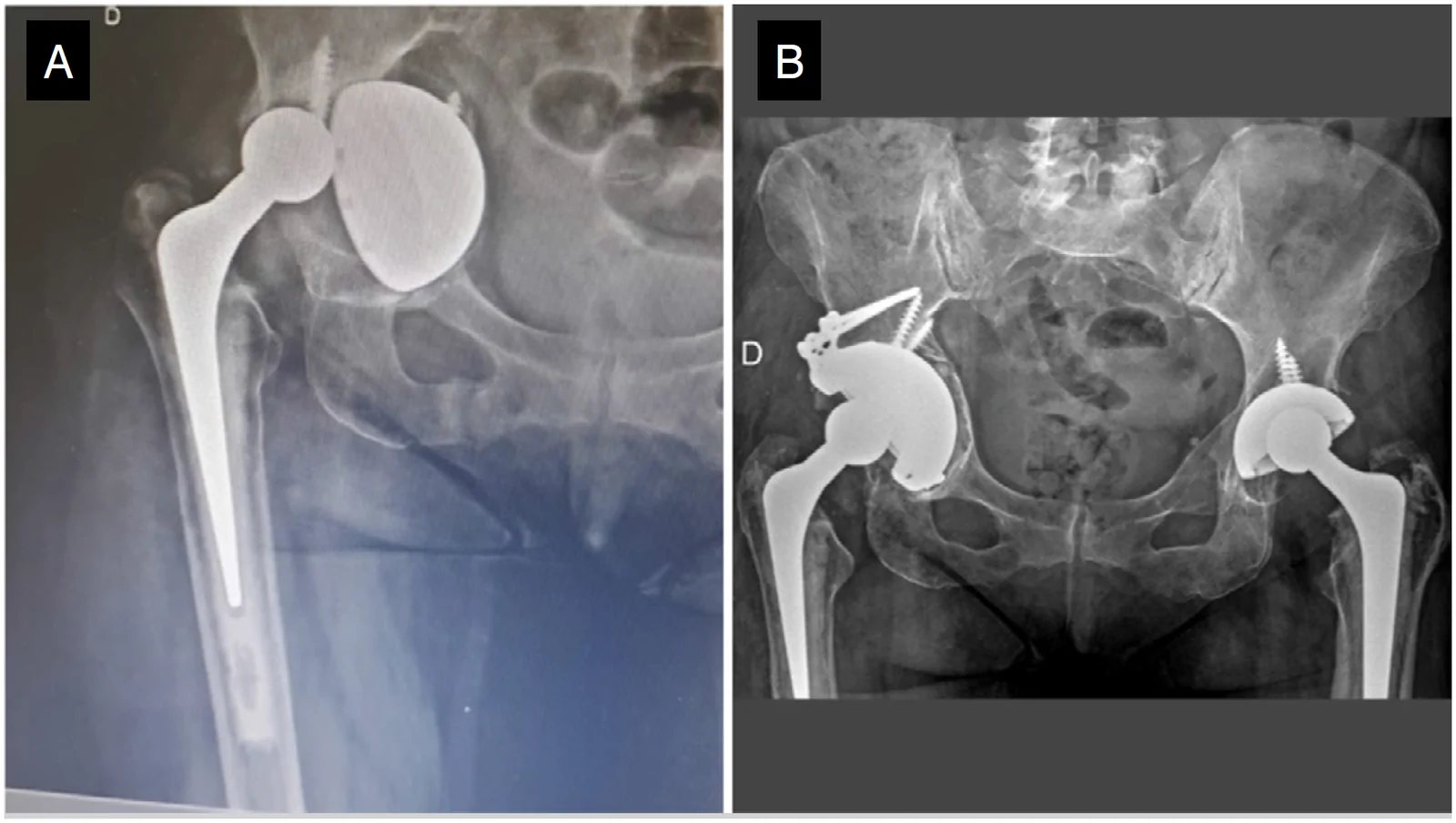
A. Preoperative radiograph of a cup-cage. B. Postoperative radiograph of a cup-cage.

The cup-on-cup technique represents an alternative for severe superior acetabular defects (Figures 3a and 3b). It is performed using two combined acetabular cups, which allows for flexibility in adapting to the bony defect and eliminates the need for prolonged preoperative planning.^1,16,17^ The majority of Brazil's public orthopedic health services have limited and restricted availability of modern implants or allograft from bone banks. The two techniques described in this study can be performed by any orthopedic surgeon, as they use materials widely available.

**Figure 3:**
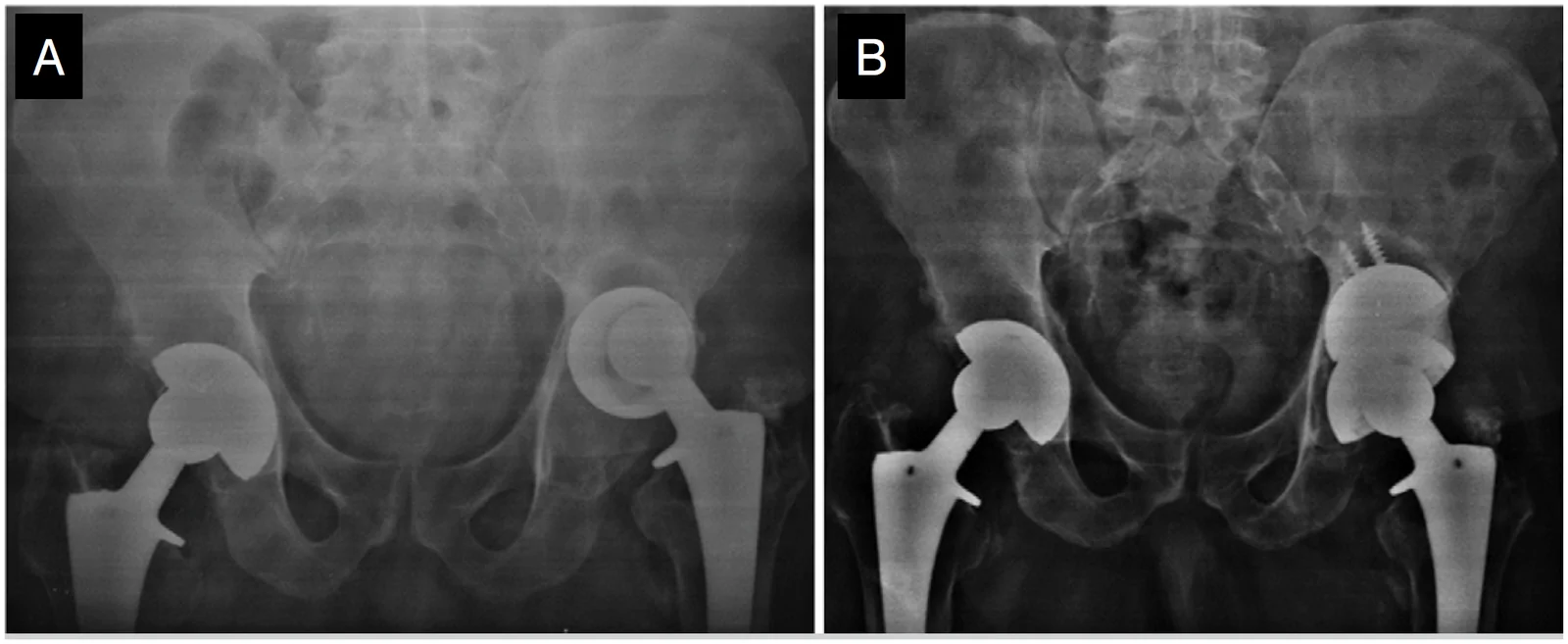
A. preoperative radiograph of a 3A acetabular defect. B. Postoperative radiograph of a cup-on-cup technique.

Despite the promising results described individually for each technique, direct comparisons between cup-on-cup and cup-cage in the same population with Paprosky defects IIC, IIIA, and IIIB remain scarce in the literature. The present study aims to describe the clinical and radiographic outcomes of these two techniques in patients undergoing acetabular revision at a hip surgery referral center.

## MATERIALS AND METHODS

This is a retrospective case series study. The surgeries were performed by orthopedic hip surgeons at the Hospital de Clínicas of the State University of Campinas (UNICAMP) between November 2017 and April 2024.

The study was approved by the Research Ethics Committee of the UNICAMP School of Medical Sciences under Opinion No. 7,094,490, CAAE: 81213724.5.0000.5404.

The authors declare that they have no conflicts of interest.

Adult patients who underwent revision of the acetabular component of total hip arthroplasty using cup-on-cup or cup-cage were included, with acetabular defects classified as Paprosky IIC, IIIA, or IIIB, and a minimum follow-up of 24 months. Patients with incomplete clinical and/or radiographic data were excluded. Ultimately, 13 cases were included in the analysis.

The bone-loss classification used for the analysis was Paprosky's, based on preoperative radiographs and confirmed intraoperatively. The criteria used to decide between cup-on-cup or cup-cage were based on intraoperative findings and the correction of specific defects. A modified cup-cage technique was used in IIC cases when jumbo cups could not provide adequate bone fixation. The cage used in the cases was made of stainless steel combined with a titanium cup. The original technique proposed by Gross, 2016 uses a titanium cage.^22^

The data collected were: age at surgery, year of surgery, Paprosky's classification, presence of acetabular osteolysis, presence of a contralateral prosthesis, degree of claudication, lower limb length discrepancy (in centimeters), hip flexion strength, postoperative range of motion (active and passive flexion, extension, abduction, and adduction, in degrees), and radiographic restoration of the hip center of rotation (assessed by anteroposterior pelvic radiography in the standing position, based on the position of the femoral head relative to the interlacrimal line and the ilioischial line on the contralateral side.

Limping was assessed using a subscore of the Harris Hip Score (HHS), a validated and widely used instrument for evaluating hip arthroplasty outcomes: Grade 0 — normal gait, completely painless and balanced; Grade I — mild claudication, detectable only by professionals; Grade II — perceptible claudication, such as a slight pelvic drop generating abnormal movement; and Grade III — pronounced and visible claudication, accompanied by a strong lateral trunk sway.

The composite factors in the primary outcome were the clinical and radiographic results of the techniques. Postoperative complications were recorded, including dislocation, periprosthetic infection, and the need for reoperation.

Descriptive statistical analysis was used, with means, standard deviations, minimum and maximum values calculated for numerical variables, and absolute and relative frequencies for categorical variables.

## RESULTS

Thirteen cases were included with a mean age of 67.5 ± 12.0 years (34–81 years). In 6 cases, the cup-cage was performed (46.2%), and in 7, the cup-on-cup (53.8%), with a mean follow-up of 54.2 ± 33.6 months (24–129 months). According to Paprosky classification, three cases presented type IIC defects (23.1%), seven type IIIA defects (53.8%), and three type IIIB defects (23.1%). At 2 years postoperatively, x-ray showed acetabular osteolysis in 10 patients (76.9%), and a contralateral prosthesis was found in 2 (15.4%). The demographic and clinical data are shown in Table 1.

**Table 1:** Demographic and clinical characteristics of the overall series.

Variable	Overall (n=13)	Cup-cage (n=6)	Cup-on-cup (n=7)
Age (years), mean ± SD	67.5 ± 12.0	70.7 ± 9.0	64.7 ± 14.3
Minimum–maximum age (years)	34–81	60–81	34–78
Follow-up (months), mean ± SD	54.2 ± 33.6	69.0 ± 40.2	41.6 ± 22.7
Min. – max. follow-up (months)	24–129	24–129	24–69
Paprosky IIC, n (%)	3 (23.1%)	3 (50.0%)	0 (0%)
Paprosky IIIA, n (%)	7 (53.8%)	1 (16.7%)	6 (85.7%)
Paprosky IIIB, n (%)	3 (23.1%)	2 (33.3%)	1 (14.3%)
Acetabular osteolysis, n (%)	10 (76.9%)	4 (66.7%)	6 (85.7%)
Contralateral prosthesis, n (%)	2 (15.4%)	0 (0%)	2 (28.6%)

Abbreviations: n, number; COR, SD, standard deviation.

Cup-cage group: 6 cases, with a mean age of 70.7 ± 9.0 years (60–81 years) and a mean follow-up of 69.0 ± 40.2 months (24–129 months). Defects were type IIC in three cases (50.0%), IIIA in one case (16.7%), and IIIB in two cases (33.3%). No cases had a contralateral prosthesis, and acetabular osteolysis was present in 4 patients (66.7%).

Cup-on-cup group: 7 cases, with a mean age of 64.7 ± 14.3 years (34–78 years) and a mean follow-up of 41.6 ± 22.7 months (24–69 months). The defects were type IIIA in 6 cases (85.7%) and type IIIB in 1 case (14.3%), with no cases of type IIC. Osteolysis was identified in 6 patients (85.7%), and a contralateral prosthesis in 2 (28.6%).

### Functional results

Mean active flexion was 88.5 ± 17.2° and passive flexion 98.5 ± 14.1°. Extension was 16.2 ± 5.1°, abduction 27.3 ± 4.4°, and adduction 30.0 ± 7.1°. In ten patients (76.9%), flexion strength was preserved; three (23.1%) had a deficit. According to the HHS limp subscore, Grade I was observed in six patients (46.2%), Grade II in four (30.8%), and Grade III in three (23.1%); in one of former, limping was attributed to contralateral hip arthritis and distal femur periprosthetic fracture with malunion (Figures 4a–4c). No patient was classified as Grade 0.

**Figure 4:**
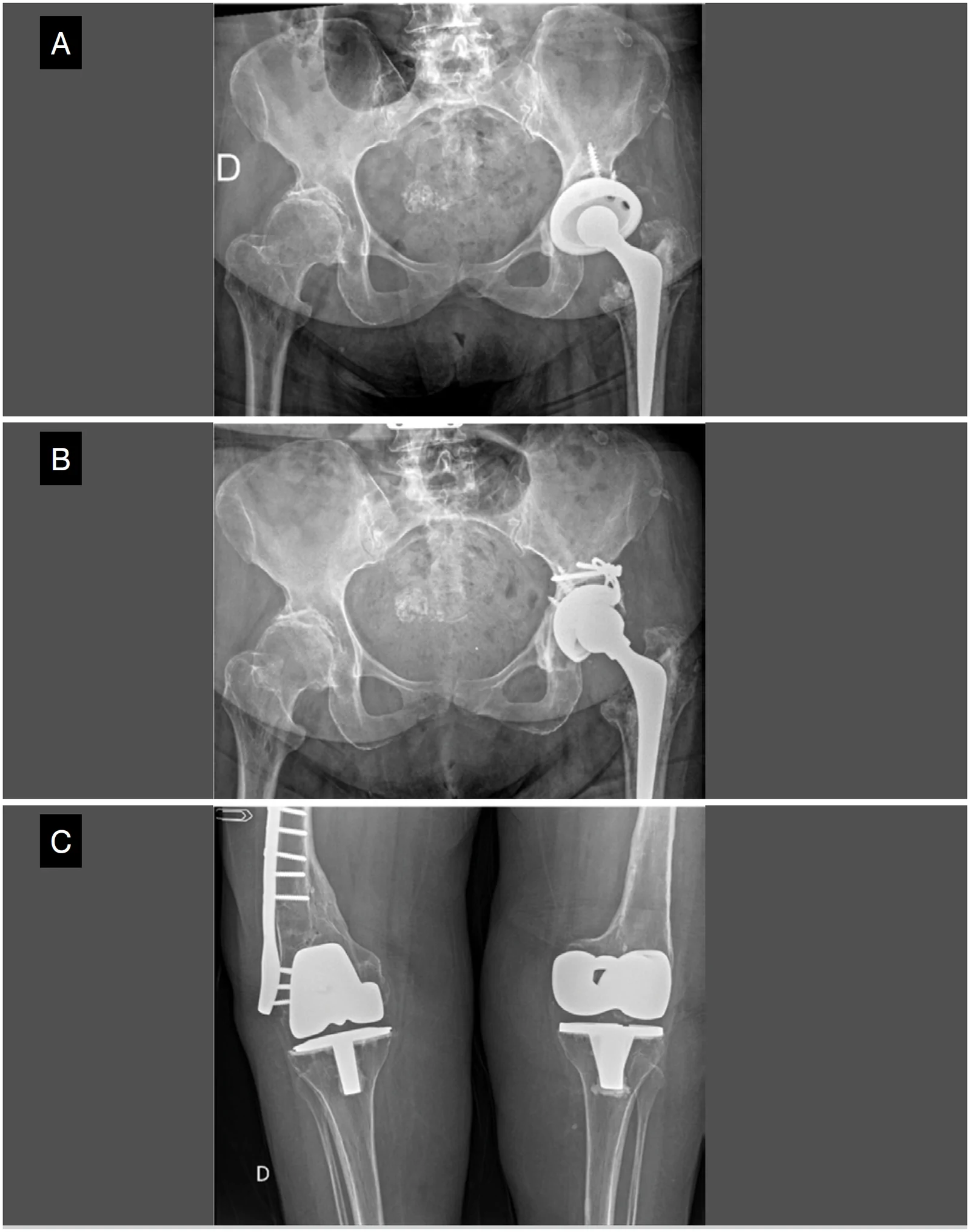
A. preoperative radiograph. B. Postoperative radiograph of a cup-cage. C. sequelae of periprosthetic femoral fracture of the knee.

Lower limb length discrepancy was identified in seven patients (53.8%, six in the cup-on-cup group and one in the cup-cage), with a mean of 0.62 ± 0.74 cm. Most cases had soft-tissue scars with superior migration, so limb length could not be restored. No significant association was found between the degree of limping and lower limb length discrepancy.

In the cup-cage group, mean active flexion was 91.7 ± 17.2°, passive flexion 101.7 ± 13.3°, extension 16.7 ± 5.2°, abduction 25.8 ± 4.9°, and adduction 28.3 ± 7.5°. Flexion strength was preserved in five cases (83.3%), and dysmetria was present in one case (16.7%), with a mean of 0.08 ± 0.20 cm. In the classification of limp, Grade I was observed in 2 patients (33.3%), Grade II in 2 (33.3%), and Grade III in 2 (33.3%).

In the cup-on-cup group, mean active flexion was 85.7 ± 18.1°, passive flexion 95.7 ± 15.1°, extension 15.7 ± 5.3°, abduction 28.6 ± 3.8°, and adduction 31.4 ± 6.9°. Preserved flexion strength was found in 5 cases (71.4%), and dysmetria was present in 6 (85.7%) - mean of 1.07 ± 0.73 cm. In the classification of limp, Grade I was observed in 4 patients (57.1%), Grade II in 2 (28.6%), and Grade III in 1 (14.3%).

### Restoration of the center of rotation

Restoration of the hip center of rotation was achieved in seven cases (53.8%); in the cup-cage group, it was achieved in four of six cases (66.7%); in the cup-on-cup group, it was achieved in three of seven cases (42.9%). One dislocation occurred in the cup-cage group (16.7%), treated with closed reduction without reoperation. No periprosthetic infection, reoperation, or prosthetic loosening was recorded. Detailed functional data are presented in Tables 2 and 3.

**Table 2:** Postoperative functional results.

Variable	Overall (n=13)	Cup-cage (n=6)	Cup-on-cup (n=7)
Active flexion (°), mean ± SD	88.5 ± 17.2	91.7 ± 17.2	85.7 ± 18.1
Passive flexion (°), mean ± SD	98.5 ± 14.1	101.7 ± 13.3	95.7 ± 15.1
Extension (°), mean ± SD	16.2 ± 5.1	16.7 ± 5.2	15.7 ± 5.3
Abduction (°), mean ± SD	27.3 ± 4.4	25.8 ± 4.9	28.6 ± 3.8
Adduction (°), mean ± SD	30.0 ± 7.1	28.3 ± 7.5	31.4 ± 6.9
Normal flexion strength, n (%)	10 (76.9%)	5 (83.3%)	5 (71.4%)
Decreased flexion strength, n (%)	3 (23.1%)	1 (16.7%)	2 (28.6%)
No asymmetry, n (%)	6 (46.2%)	5 (83.3%)	1 (14.3%)
Asymmetry present, n (%)	7 (53.8%)	1 (16.7%)	6 (85.7%)
Asymmetry (cm), mean ± SD	0.62 ± 0.74	0.08 ± 0.20	1.07 ± 0.73

Abbreviations: n, number; cm, centimeter.

**Table 3:** Limping and restoration of the center of rotation.

Variable	Overall (n=13)	Cup-cage (n=6)	Cup-on-cup (n=7)
Limp Grade I, n (%)	6 (46.2%)	2 (33.3%)	4 (57.1%)
Limp Grade II, n (%)	4 (30.8%)	2 (33.3%)	2 (28.6%)
Limp Grade III, n (%)	3 (23.1%)	2 (33.3%)	1 (14.3%)
Restoration of the COR — Yes, n (%)	7 (53.8%)	4 (66.7%)	3 (42.9%)
COR restoration — No, n (%)	6 (46.2%)	2 (33.3%)	4 (57.1%)
Dislocation, n (%)	1 (7.7%)	1 (16.7%)	0 (0%)
Periprosthetic infection, n (%)	0 (0%)	0 (0%)	0 (0%)
Reoperation, n (%)	0 (0%)	0 (0%)	0 (0%)

Abbreviations: n, number; COR, center of rotation.

## DISCUSSION

Treating Paprosky IIC, IIIA, and IIIB acetabular defects remains one of the greatest challenges in hip revision arthroplasty.^4,7,8^ These defects require careful planning, surgical expertise, and specific implants. This study describes the experience of a referral center with 13 patients who underwent surgery using the cup-cage and cup-on-cup techniques, followed for a mean of 54.2 months, with 1 case of dislocation treated with closed reduction. There were no other complications or reoperations during follow-up.

Chiarlone et al. obtained similar results in their case series, with no cases of aseptic loosening or dislocation.^18^ Systematic reviews on cup-on-cup arthroplasty report dislocation rates of around 9% and infection rates of around 4%, with an overall complication rate of 20%.^14^ With 131 cases and 7.7 years of follow-up, Chaudhry et al. documented dislocation in 7.6% and infection in 9.1%.^19^ In contrast, Chen et al. did not identify aseptic loosening over more than ten years of follow-up—dislocation and infection each occurred in 7% of cases. ^15^ In the study by Cano et al. involving the reconstruction of Paprosky type III acetabular defects using reconstruction rings, a similar complication profile was observed; no intraoperative complications were recorded, but there was a 17.39% rate of postoperative dislocation, all cases of which were resolved via closed reduction.^20^

These findings are consistent with the present study, despite the small sample size. Hip center of rotation restoration was achieved in 53.8% of cases in the overall series, with better performance in the cup-cage group (66.7%) compared to the cup-on-cup group (42.9%). This is considered a fundamental goal of acetabular revision surgery, as it directly impacts abductor muscle function and implant stability/longevity.^1,12^ Du et al. reported correction of superior displacement of the center of rotation from 60.9 mm to 31.7 mm in cup-on-cup cases for IIIB defects.^16^ Webb et al., however, demonstrated a reduction from 55.7 mm to 22.5 mm in a series of 20 cases using the cup-on-cup technique.^17^ The lower rate of satisfaction observed in the cup-on-cup group in this study may reflect the greater complexity of the defects treated in this group, predominantly IIIA (85.7%), with no cases of IIC defects, compared to the cup-cage group, where IIC defects were more frequent.

In the cup-on-cup group, there was a higher prevalence of vertical elevation of the center of rotation (85.7% of cases, mean 1.07 cm) than in the cup-cage group (16.7%, mean 0.08 cm). This difference is likely related to the greater severity of the acetabular defects treated by the cup-on-cup technique in this study, as well as to the limitations inherent in restoring femoral offset in defects with significant superior migration of the center of rotation.^12,14^ In the study by Chaudhry et al., a significant improvement in dysmetria from 12.2 mm to 3.9 mm was documented with the cup-cage,^19^ reinforcing that the appropriate choice of technique, guided by the severity of the defect, is crucial for correcting this parameter. Intraoperative soft-tissue contracture and superior migration due to bone loss were identified as the primary limiting factors when length discrepancy was not achieved.

Both groups exhibited very similar ranges of motion, with no significant difference between the groups. Both groups exhibited very similar ranges of motion, with no significant difference between the groups. Flexion strength remained normal in most patients, but in three (23.1%) that may be attributed to iliopsoas dysfunction, as this muscle is vulnerable in hips with significant superior migration of the center of rotation and a history of multiple previous surgical interventions^4,8^

We found encouraging data in the literature regarding the cup-on-cup technique and cup-cage in studies with long-term follow-up. In a study by Chen et al., a 10-year survival rate of 90% was observed (2), and Chaudhry et al. reported a 95.5% survival rate for aseptic loosening.^19^ For the cup-on-cup technique, follow-up data are even more limited, with a shorter average follow-up period of less than 5 years.^1,16,17^ In one of the largest comparative studies, involving 180 cases of Paprosky IIIA and IIIB, Tarabichi et al. did not identify a statistically significant difference in survival between the techniques and implants. This study evaluated the rates of dislocation or infection among porous metal wedges, cup-cages, and custom triflanged implants, including cases with pelvic discontinuity^21^, suggesting that the surgeon's experience and the appropriateness of the technique to the specific defect are determining factors for outcome.

Regarding the study's limitations, we note the small sample size and the retrospective design, which preclude a more comprehensive comparative statistical analysis between the techniques. The heterogeneity in the distribution of cases across classification and technique type also limits the comparative analysis between the cup-cage and the cup-on-cup technique. The absence of functional scores, such as the Harry Hip Score, also presents an additional limitation. Future studies with larger sample sizes and longer follow-up periods are needed.

## CONCLUSION

Cases treated with cup-on-cup and cup-cage showed promising functional and radiographic outcomes. The center of rotation was restored more frequently in the cup-cage. Dysmetria, however, was more common in the cup-on-cup group, likely related to the severe bone loss defects predominant in that group. Mobility and flexion strength were preserved in most patients in both groups.

No single technique is absolutely superior to another. The choice of technique depends on the defect present, the surgeon's experience, and the implant available at the time of surgery. The techniques evaluated in this study represent good alternatives given the limitations in material availability found in most public health care systems. The study is particularly relevant in underdeveloped countries, where limited access to more expensive implants makes “cup-on-cup” and “cup-on-cage” techniques viable alternatives, enabling the resolution of complex acetabular defects.

## Data Availability

The contents underlying the research are available in the manuscript.
